# Selective androgen receptor modulators (SARMs) have specific impacts on the mouse uterus

**DOI:** 10.1530/JOE-19-0153

**Published:** 2019-07-18

**Authors:** Ioannis Simitsidellis, Arantza Esnal-Zuffiaure, Olympia Kelepouri, Elisabeth O’Flaherty, Douglas A Gibson, Philippa T K Saunders

**Affiliations:** 1 Centre for Inflammation Research, The University of Edinburgh, Queen’s Medical Research Institute, Edinburgh BioQuarter, Edinburgh, UK

**Keywords:** uterus, endometrium, androgen, SARM, Andarine, GTx-024, Enobosarm, Ostarine, Danazol, DHT

## Abstract

Selective androgen receptor modulators (SARMs) have been proposed as therapeutics for women suffering from breast cancer, muscle wasting or urinary incontinence. The androgen receptor (AR) is expressed in the uterus but the impact of SARMs on the function of this organ is unknown. We used a mouse model to compare the impact of SARMs (GTx-007/Andarine®, GTx-024/Enobosarm®), Danazol (a synthetic androstane steroid) and dihydrotestosterone (DHT) on tissue architecture, cell proliferation and gene expression. Ovariectomised mice were treated daily for 7 days with compound or vehicle control (VC). Uterine morphometric characteristics were quantified using high-throughput image analysis (StrataQuest; TissueGnostics), protein and gene expression were evaluated by immunohistochemistry and RT-qPCR, respectively. Treatment with GTx-024, Danazol or DHT induced significant increases in body weight, uterine weight and the surface area of the endometrial stromal and epithelial compartments compared to VC. Treatment with GTx-007 had no impact on these parameters. GTx-024, Danazol and DHT all significantly increased the percentage of Ki67-positive cells in the stroma, but only GTx-024 had an impact on epithelial cell proliferation. GTx-007 significantly increased uterine expression of *Wnt4* and *Wnt7a*, whereas GTx-024 and Danazol decreased their expression. In summary, the impact of GTx-024 and Danazol on uterine cells mirrored that of DHT, whereas GTx-007 had minimal impact on the tested parameters. This study has identified endpoints that have revealed differences in the effects of SARMs on uterine tissue and provides a template for preclinical studies comparing the impact of compounds targeting the AR on endometrial function.

## Introduction

Androgens are pleiotropic hormones which bind with high affinity and specificity to androgen receptors (ARs) to regulate both reproductive and other tissues. In the uterus, androgen-target cells include stromal fibroblasts and epithelial cells surrounding the lumen and glands of the endometrium, as well as the smooth muscle cells of the myometrium (Simitsidellis *et al.* 2018). Studies in women and rodents have demonstrated that secretions from endometrial glands play a fundamental role in the establishment of pregnancy in both species (Filant & Spencer 2013, Spencer 2014, Kelleher *et al.* 2018). In response to ovarian hormones endometrial stromal cells differentiate into specialised secretory decidual cells which provide critical nutritional support to the early embryo and play a key role in regulation of trophoblast invasion and placental formation (Gellersen & Brosens 2014).

Over the past 20 years, there has been an increase in efforts to develop compounds that act via AR to promote and maintain the anabolic impacts of androgens on muscle and bone but without the undesirable side effects associated with exogenous androgen administration, including prostate hyperplasia in men or virilisation in women. The result of these efforts has been the synthesis of a new class of nonsteroidal drugs known as selective androgen receptor modulators (SARMs; Gao & Dalton 2007, Mohler *et al.* 2009, McEwan 2013, Dalton 2017). In the current paper we have used a mouse model to test the impact of two SARMs, GTx-007 (also known as Andarine, S4: https://pubchem.ncbi.nlm.nih.gov/compound/9824562) and GTx-024 (Enobosarm, Ostarine: https://pubchem.ncbi.nlm.nih.gov/compound/11326715) on uterine tissue and to compare their activities with the potent, natural, non-aromatisable androgen dihydrotestosterone (DHT). The model chosen was one in which we have previously identified changes in gene expression and tissue function in response to DHT (Simitsidellis *et al.* 2016). Briefly, ovariectomised mice were administered either a single subcutaneous injection or seven daily injections of DHT (0.2 mg/mouse). Treatment with DHT resulted in a time-dependent increase in uterine size, characterised by an early wave of epithelial cell proliferation, change in the expression of genes encoding factors involved in stromal–epithelial cross-talk and a significant increase in the number of uterine glands in samples recovered on day 7 of treatment (Simitsidellis *et al.* 2016). In the current study, we also compared the impact of SARMs to Danazol (https://pubchem.ncbi.nlm.nih.gov/compound/danazol), an orally active synthetic steroid derived from ethinyl testosterone, which is able to bind to AR and sex hormone-binding globulin (Barbieri & Ryan 1981). Danazol has been used since the 1970s to treat women with endometriosis, as well as other conditions, including pain associated with benign fibrocystic breast disease (Ramsey-Stewart 1988). Although Danazol was reported to be an effective treatment for endometriosis-associated pain, androgenic side effects (acne, deepening of voice, hirsutism) have limited its use (Selak *et al.* 2007).

The development of a new generation of SARMs reported to have high anabolic and low androgenic activities has renewed interest in using them as therapeutics in women with muscle wasting, urinary incontinence and breast cancer. There have been a number of promising preclinical studies in rodents as well as some clinical trials assessing the effects of SARMs on muscle and bone. Specifically, in rodent models, GTx-024 has been reported to (1) restore pelvic floor muscle weight of ovariectomised mice to sham levels (Ponnusamy *et al.* 2017), (2) increase bone mineral content, bone mineral density and bone volume density in a rat osteoporosis model (Hoffmann *et al.* 2019) and (3) restore levator ani muscle weight of orchidectomised mice to sham levels (Dubois *et al.* 2015). A phase II randomised, double-blind clinical trial assessing the efficacy of GTx-024 in cancer patients with muscle cachexia demonstrated a significant increase in total lean body mass of patients in the GTx-024 group compared to placebo (Dobs *et al.* 2013). Clinical trials assessing the efficacy of GTx-024 for urinary incontinence (NCT03241342) and AR-positive triple-negative breast cancer (NCT01616758) are complete and release of results was pending at the time this manuscript was written.

GTx-007 (or Andarine) was reported to be a partial AR agonist able to restore total lean body mass, soleus and levator ani muscle mass of castrated male rats to those of intact animals, with minimal stimulation of the prostate (Gao *et al.* 2005). Moreover, GTx-007 significantly reduced ovariectomy-induced bone loss of female rats (Kearbey *et al.* 2007) and induced a significant increase in total body bone mineral density, as measured by dual X-ray absorptiometry in castrated male rats (Gao *et al.* 2005).

Danazol has been used as a treatment for endometriosis due to its ability to generate a high androgen and low oestrogen environment, thus resulting in the atrophy of endometriotic implants and its use as a therapeutic agent has been demonstrated by its effectiveness in reducing endometriosis-associated pain and laparoscopic scores (Selak *et al.* 2007). A Cochrane systematic review comparing the effects of various treatments for endometriosis to those of gonadotrophin‐releasing hormone analogues (GnRHas) on the bone mineral density (BMD) of women with endometriosis concluded that between the groups receiving GnRHa and the groups receiving Danazol, there was a significant difference in percentage change of BMD after 6 months of treatment, with the GnRH analogue producing a reduction in BMD from baseline and Danazol producing an increase in BMD (Sagsveen *et al.* 2003).

The endometrium and myometrium in women and mice both express AR, however, little is known about the impact of SARMs on the uterus. GTx-024 was reported to significantly increase uterine wet weight in ovariectomised rats (Hoffmann *et al.* 2019) but to the best of our knowledge the impact of GTx-007 on the uterus has not been investigated to date. In the current study we used a previously validated mouse model to assess the impact of SARMs on the uterus. The primary aim of this study was to explore changes in uterine tissue in response to treatment using a combination of immunohistochemistry, high-throughput image analysis and analysis of androgen-regulated candidate genes to identify any uterine-specific impacts of SARMs.

## Methods

### Animals and treatments

Female C57BL/6J mice were purchased from Charles River Laboratories and allowed to acclimate for a week with *ad libitum* access to food and water. Experiments were performed under a licence granted by the UK Home Office (PPL 70/8945) and were approved by the University of Edinburgh Animal Welfare and Ethical Review Body. A previously established protocol was used to assess the impact of androgens (Simitsidellis *et al.* 2016). Briefly, 8- to 10-week-old mice were ovariectomised by dorsal bilateral ovariectomy and allowed to recover for 7 days prior to treatment, to deplete endogenous sex-steroid hormones. Surgery was performed under isoflurane anaesthesia followed by a post-operative analgesic, buprenorphine (0.1 mg/kg), for pain management. Ovariectomised mice were randomly assigned into one of five treatment groups (*n* = 10–14 per treatment group) and received seven daily subcutaneous injections of either vehicle control (VC; 5% ethanol, 0.4% methylcellulose), DHT (0.2 mg/mouse), GTx-007 (Andarine; 0.5 mg/mouse), GTx-024 (Ostarine; 0.5 mg/mouse) or Danazol (1.25 mg/mouse). At the time of tissue recovery, body weight and weights of uterine tissue were recorded. One uterine horn was fixed in 4% neutral buffered formalin (NBF) overnight at room temperature and the other horn was placed in RNA Save® (GeneFlow, Lichfield, UK) and stored at −80°C.

### Histology and immunohistochemistry

Uterine tissue samples were processed according to standard procedures: transverse sections of 5 μm thickness were either stained with H&E or specific antibodies (Table 1) using methods detailed in Simitsidellis *et al.* (2016). Detection was performed using a polymer-based detection system (ImmPRESS, Vector Labs) using the chromogen 3,3′-diaminobenzidine (DAB) (Vector) as per the manufacturer’s instructions. Between incubations, washes were performed with TBS-Tween. Haematoxylin was used as a counterstain.

**Table 1:** Details of antibodies used for immunohistochemistry.

Protein target	Antibody	Supplier	Catalogue number	Species raised in	Dilution
Androgen receptor	AR	Spring Bioscience	M4070	Rabbit monoclonal	1/600
Marker of proliferation Ki67	MKi67	Abcam	Ab15580	Rabbit polyclonal	1/2000
Forkhead box protein 2	FOXA2	Santa Cruz	SC9187	Goat polyclonal	1/1000

### Image acquisition and high-throughput image analysis

Stained slides were scanned using an Axio Scan.Z1 Slidescanner (Zeiss). For quantitative image analysis, a minimum of two non-serial sections per animal were used (*n* = 8–14 animals/treatment group), with at least 50 μm distance between each cut section. High-throughput image analysis was performed using the StrataQuest v5.0 software (TissueGnostics, Vienna, Austria). Briefly, the software unmixes two markers (chromogen and counterstain) and segments single cells into nuclei, perinuclear areas and cytoplasm (http://www.tissuegnostics.com/en/products/analysing-software/strataquest). Each segmented cell compartment is measured for up to 20 intensity, statistic and morphometric parameters which are displayed in scattergrams and histograms. For DAB-stained sections, negative control slides (omission of primary antibody) were used to set the threshold of detection. Quantification of FOXA2-positive endometrial glands was performed by blinded manual counting.

### RNA extraction and reverse transcription

Total RNA was extracted from homogenised mouse uterine tissue (20 mg per sample) using standard methods. RNA concentration and quality were measured using a NanodropND-1000 spectrophotometer (Nanodrop Technologies): samples were standardised to 100 ng/μL in RNAse-free water. Reverse transcription was performed using the SuperScript VILO cDNA Synthesis Kit (Invitrogen) as per the manufacturer’s instructions using a thermal cycler programmed at 25°C for 10 min, 42°C for 60 min and 52°C for 5 min. Two negative controls (omission of reverse transcriptase control and omission of RNA control) were included for each set of RNA samples and pooled RNA from all samples was used to generate a ten-fold serial dilution set of standards for standard curve analysis.

### Quantitative real-time PCR analysis

Quantitative real-time PCR (TaqMan method) was performed using SuperMix with Premixed ROX dye (Invitrogen), primer sets were designed using the Roche Universal Probe Library Assay Design Centre and purchased from Eurofins MWG Operon (Ebersberg, Germany) and probes from the Roche Universal Probe Library Mouse Set (Roche Applied Science). Samples were assayed in duplicate and run on an ABI 7900HT Fast Real-Time PCR machine using the following conditions: 95°C for 10 minutes then 40 cycles of 95°C for 15 s and 60°C for 1 min. Primer amplification efficiency was validated, and analysis was performed using the relative standard curve method. Data were normalised to *Actb* and fold-change is expressed as the ratio of expression of each gene of interest in the treated groups against the average of the VC groups. Statistical analysis was performed using GraphPad Prism 7.0. Data are presented as mean ± s.e.m. and statistical comparisons are described in figure legends. Criterion for significance was *P* < 0.05. Primer pair and probe information is provided in Supplementary Table 1 (see section on supplementary data given at the end of this article).

## Results

### Uterine morphometric parameters are influenced by AR modulation

**Figure 1:**
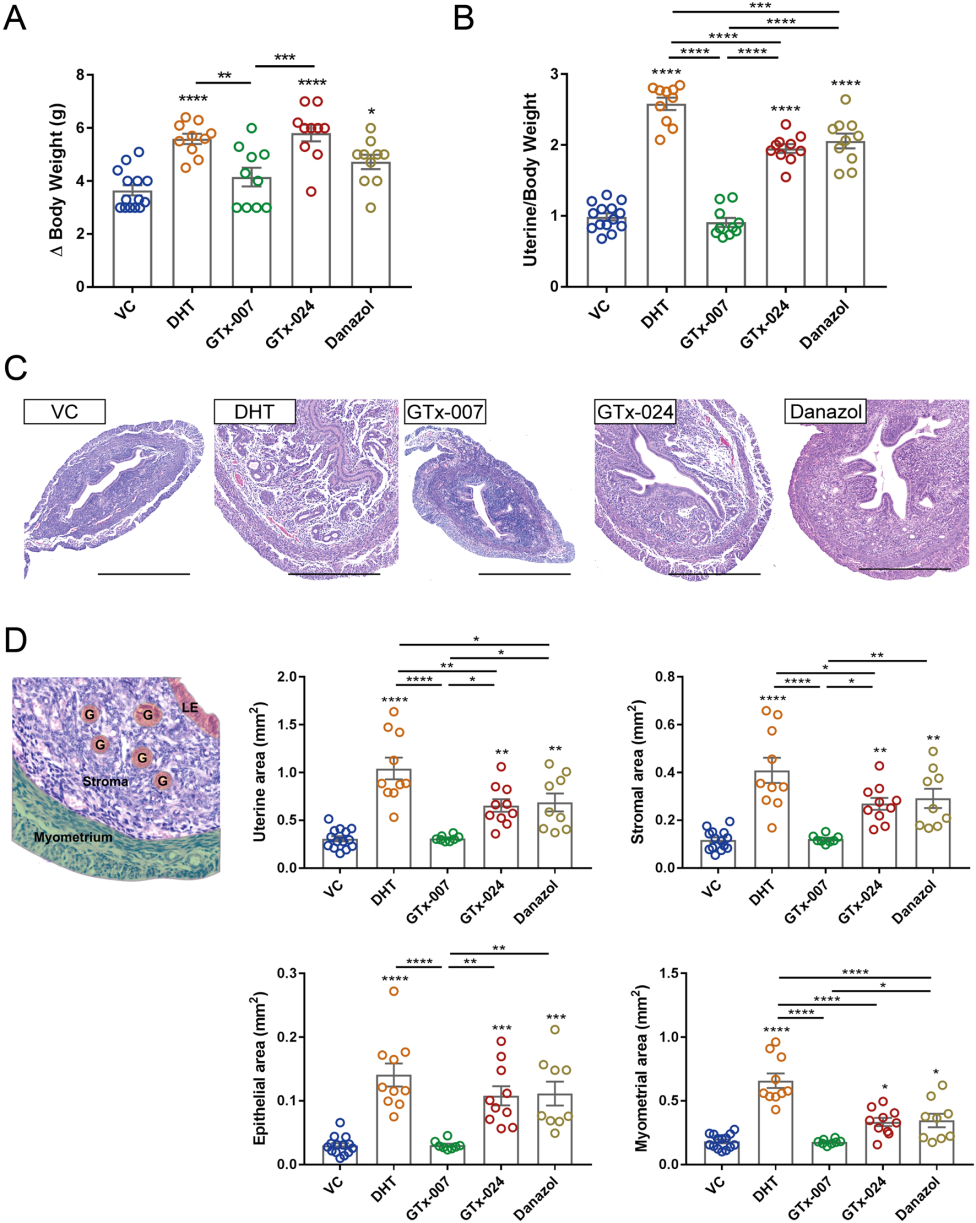
Compartment-specific changes in morphometric parameters induced by AR modulation in the mouse uterus. Female C57BL/6J mice were ovariectomised and treated with AR ligands as described in the ‘Materials and methods’ section. (A) Changes in total body weight of treated mice between the time of ovariectomy and tissue collection. (B) Changes in uterine weight normalised to animal weight following treatments. (C) Representative H&E uterine cross-sections of treated mice are shown, with increases in uterine size being accompanied by enlargement of individual cells. (D) Cross-section of a mouse uterus stained with H&E. Uterine cellular compartments are highlighted (myometrium, stroma, glands (G) and luminal epithelium (LE)). Analysis of surface area (in mm^2^) of the uterus (myometrium + endometrium), the stromal compartment, the epithelial compartment (glandular + luminal) and the myometrium following treatments. *n* = 10–14/treatment group. One-way ANOVA with Tukey’s multiple comparisons test was used for comparisons between treatment groups. Plain stars (*) indicate comparisons with VC, while stars above lines demonstrate comparisons between indicated treatment groups (*P < 0.05, **P < 0.01, ***P < 0.001, ****P < 0.0001). Scale bars: 500 μm. DHT, dihydrotestosterone; VC, vehicle control.

Endogenous hormones were depleted by ovariectomy before the start of treatments. In mice that received seven daily injections of DHT, GTx-024 or Danazol, significant increases in both body weight (Fig. 1A) and uterine weight (Fig. 1B) were detected compared to VC-treated mice, while no significant difference was detected in the GTx-007-treated group. On H&E-stained tissue sections, the impact of ovariectomy and treatments on overall uterine architecture was apparent (Fig. 1C). Consistent with depletion of ovarian hormones, ovariectomy resulted in a reduction in the cross-sectional surface area of the uterus, as well as the area occupied by the endometrial stroma and epithelium in the vehicle treatment group (VC) (Fig. 1C). Treatment with DHT, GTx-024 and Danazol all appeared to increase uterine area (Fig. 1C), while the uterine area in GTx-007-treated mice was not different from VC.

To quantify compartment-specific changes in the uteri of mice treated with AR modulators, image analysis was performed using the StrataQuest v5.0 software, which excluded luminal area and tissue gaps. Uterine surface area was significantly increased following treatment with DHT, GTx-024 and Danazol (Fig. 1D) mirroring the changes in uterine weight (Fig. 1B). The changes in total uterine surface area were reflected in all compartments, with DHT, GTx-024 and Danazol inducing expansion of the stromal, epithelial (luminal and glandular) and myometrial compartments (Fig. 1D). In contrast, treatment for 7 days with GTx-007 resulted in uterine architecture and compartment measurements which appeared unchanged compared with VC (Fig. 1D).

### Compartment-specific changes in AR expression induced by AR modulation

**Figure 2:**
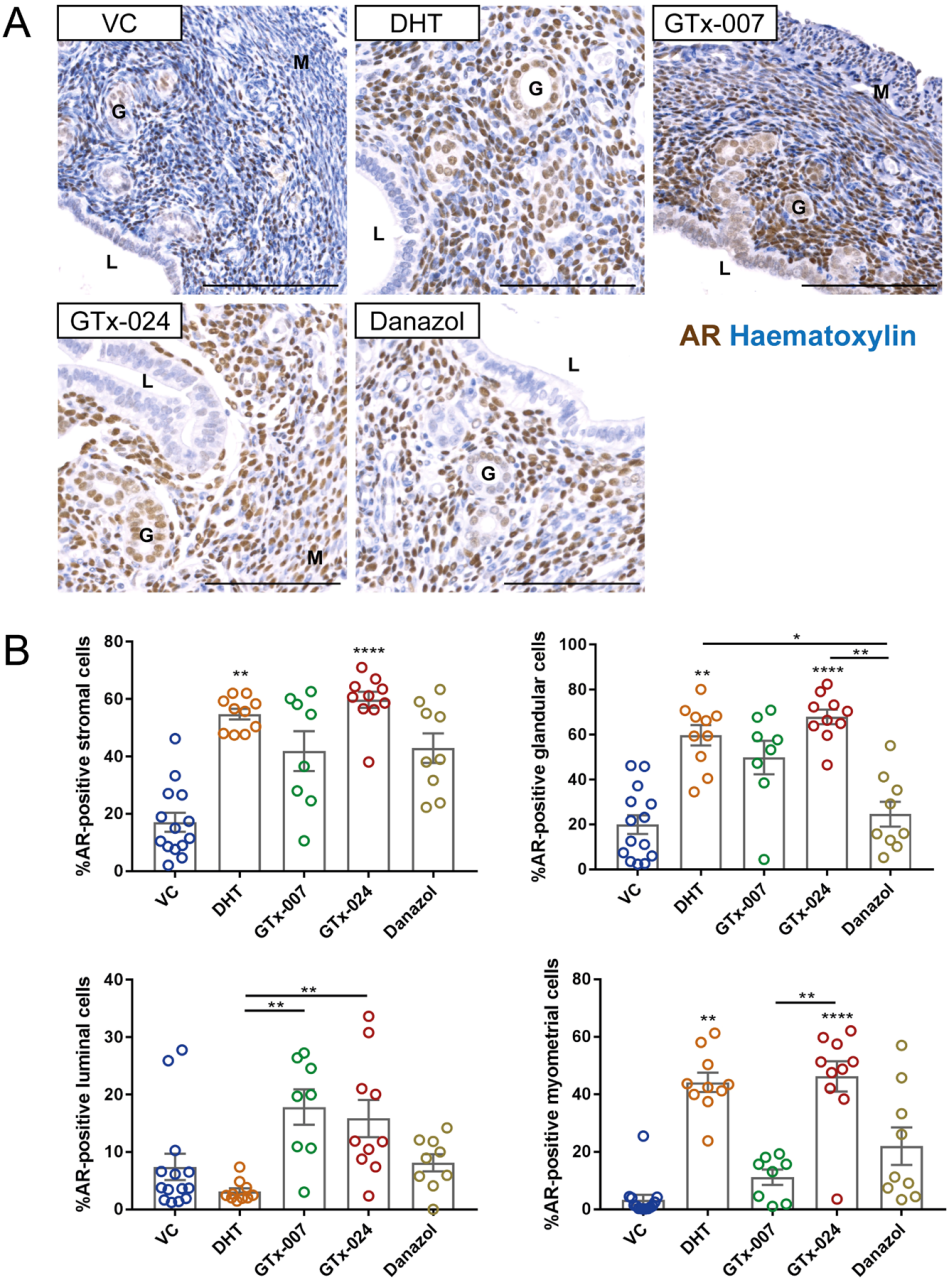
Treatment with the SARM GTx-024 altered AR protein levels in the uterus. (A) Uterine cross-sections of treated mice were stained with immunohistochemistry for AR (brown) and were counterstained with haematoxylin (blue). Uterine compartments are indicated (luminal epithelium (L), glandular epithelium (G) and myometrium (M)). Scale bars: 100 μm. (B) High-throughput image analysis of AR-stained sections using StrataQuest revealed a significant increase in the percentage of AR-positive cells in all uterine compartments following treatment with GTx-024 compared to VC. DHT upregulated AR in the stroma, glands and myometrium, while uterine sections of GTx-007- and Danazol-treated mice exhibited no change in the percentage of AR-positive cells compared to VC treatment. *n* = 8–14/treatment group. Kruskal–Wallis with Dunn’s multiple comparisons test was used for comparisons between treatment groups. Plain stars (*) indicate comparisons with VC, while stars above lines demonstrate comparisons between indicated treatment groups (*P < 0.05, **P < 0.01, ****P < 0.0001). DHT, dihydrotestosterone; VC, vehicle control.

Protein levels of AR in the uteri of treated mice were analysed by high-throughput quantitative image analysis (Fig. 2A and B). Mice treated with VC exhibited low AR expression in all uterine compartments, with only 20% of stromal and glandular cells being AR positive. Treatment with DHT or GTx-024 significantly increased the number of AR-positive cells in the stroma, the glands and the myometrium, while Danazol-treated mice displayed no change in the percentage of AR-positive cells compared to VC in the stroma, epithelium or myometrium. Treatment with GTx-007 resulted in a highly heterogeneous pattern of AR expression in all compartments, with a significant increase of AR-positive cells in the luminal epithelium compared to VC.

### Compartment-specific changes in cellular proliferation induced by AR modulation

**Figure 3:**
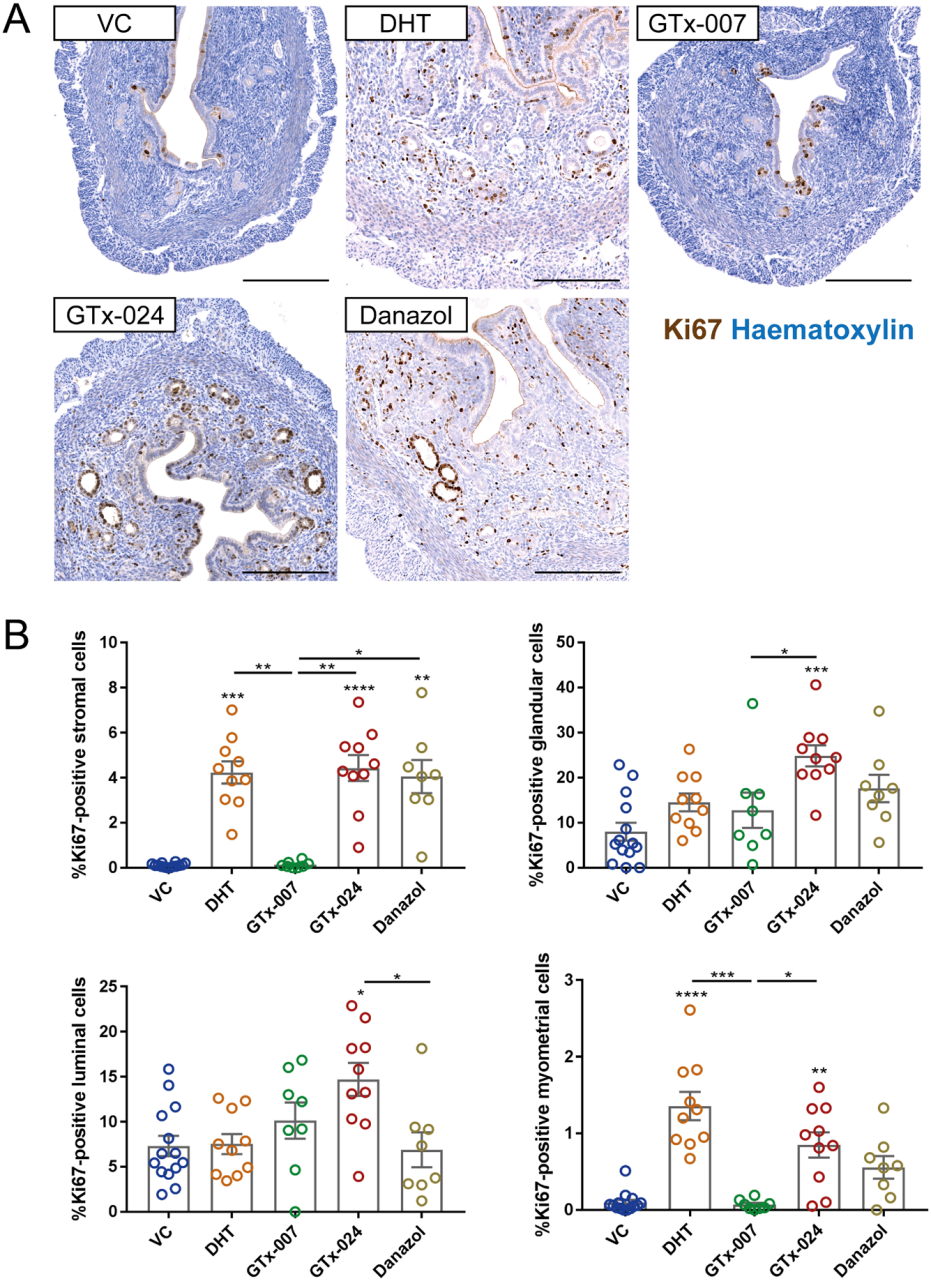
Compartment-specific effects of SARM on uterine cellular proliferation. (A) Uterine cross-sections of treated mice were stained with immunohistochemistry for the proliferation marker Ki67 (brown) and were counterstained with haematoxylin (blue). Scale bars: 200 μm. (B) High-throughput image analysis of Ki67-stained sections using StrataQuest revealed a significant increase in the percentage of Ki67-positive cells in the stromal compartment following treatment with DHT, GTx-024 and Danazol. GTx-024 treatment induced a significant increase in the percentage of Ki67-positive cells in the glands and the myometrium, with DHT only partially mirroring this effect. *n* = 8–14/treatment group. Kruskal–Wallis with Dunn’s multiple comparisons test was used for comparisons between treatment groups. Plain stars (*) indicate comparisons with VC, while stars above lines demonstrate comparisons between indicated treatment groups (*P < 0.05, **P < 0.01, ***P < 0.001, ****P < 0.0001). DHT, dihydrotestosterone; VC, vehicle control.

The percentage of proliferating cells in uterine compartments was analysed by Ki67 immunostaining followed by high-throughput quantitative image analysis (Fig. 3A and B). Expression of Ki67 was almost undetectable in the stromal compartment and myometrium of VC- and GTx-007-treated mice, while basal levels of Ki67 expression (approximately 5–10% of cells) were readily detectable in the luminal and glandular epithelial compartments. Treatment with DHT, GTx-024 and Danazol all significantly increased the percentage of Ki67-positive cells in the stromal compartment. In addition, GTx-024 significantly increased the percentage of Ki67-positive cells in the glandular and luminal epithelium, as well as in the myometrium compared to VC. Notably, the percentage of proliferating cells in the stroma and myometrium did not exceed 5% under any of the treatment conditions.

### GTx-024 and Danazol induce an increase in endometrial glands

**Figure 4:**
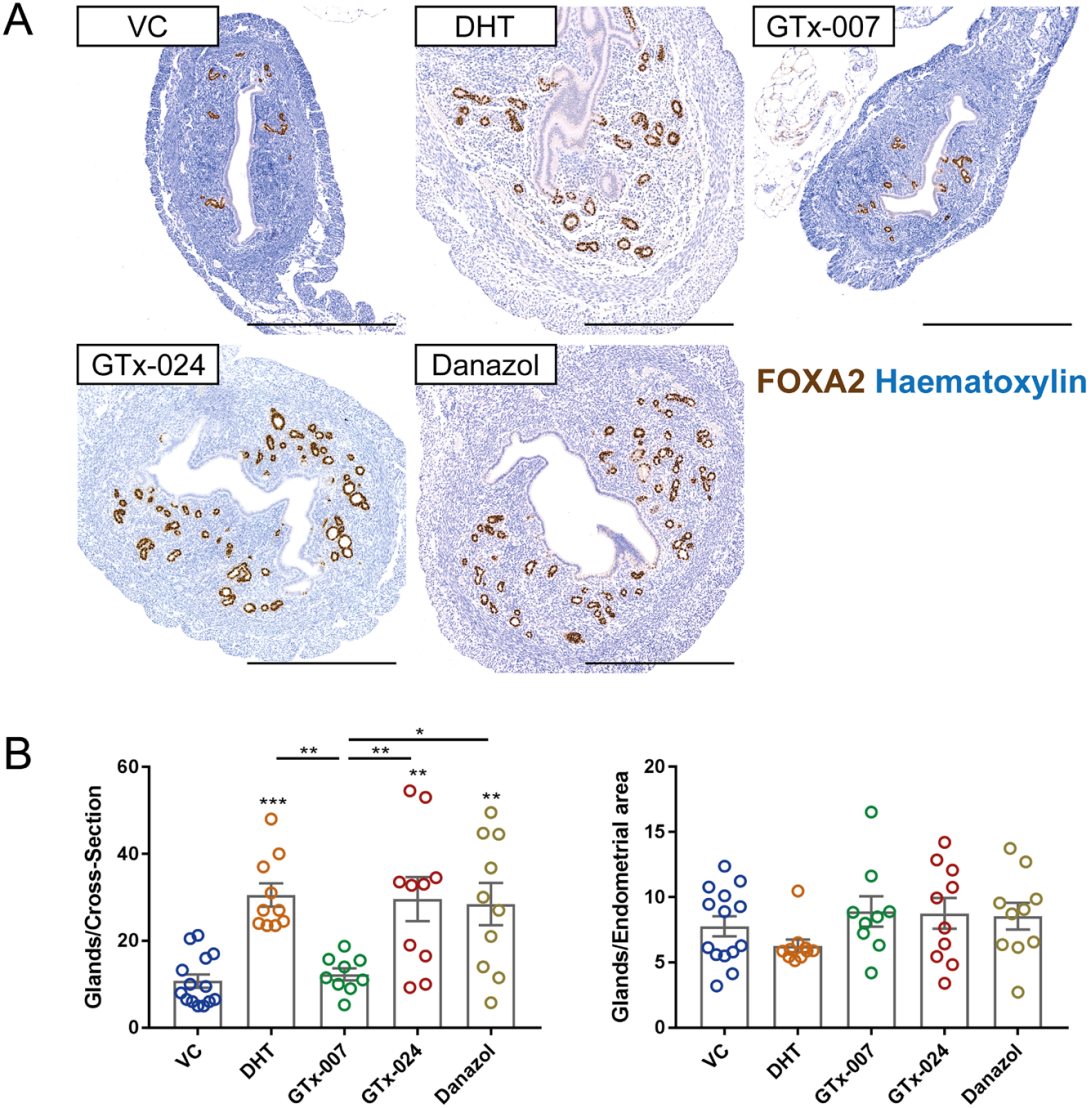
Uterine stimulation by DHT, GTx-024 and Danazol induces glandular expansion. (A) Uterine cross-sections of treated mice were stained with immunohistochemistry for the gland-specific transcription factor Foxa2 (brown) and were counterstained with haematoxylin (blue). Scale bars: 500 μm. (B) Quantification of endometrial glands identified significant increase in the absolute number of glands in the endometrium of mice treated with DHT, GTx-024 and Danazol. Normalisation to endometrial area (epithelial + stromal) revealed that the density of endometrial glands is unchanged following treatments. *n* = 9–14/treatment group. One-way ANOVA with Tukey’s multiple comparisons test was used for comparisons between treatment groups. Plain stars (*) indicate comparisons with VC, while stars above lines demonstrate comparisons between indicated treatment groups (*P < 0.05, **P < 0.01, ***P < 0.001). DHT, dihydrotestosterone; VC, vehicle control.

Elegant studies in knockout mice have identified FOXA2 as a critical endometrial gland-specific transcription factor and confirmed the importance of gland number in establishment and maintenance of pregnancy (Kelleher *et al.* 2018). To complement and extend our previous study, in which we detected an increase in FOXA2-positive glands/cross-section in mice treated with DHT for 7 days, we quantified numbers of glands in all treatment groups (Fig. 4A and B). Mice treated with either VC or GTx-007 had approximately ten glands per uterine cross-section within an endometrium, with a compact stroma, consistent with overall endogenous steroid depletion (Fig. 4B). Treatment with DHT, GTx-024 or Danazol increased the number of glands compared to the VC treatment group by an average three-fold. We had already determined that the area of the endometrial stroma was also significantly increased by these treatments (Fig. 1D) and consistent with this, the number of glands/endometrial area was similar between groups.

### Gene expression analysis of transcripts involved in stromal–epithelial cross-talk in the mouse uterus following treatment with AR modulators

**Figure 5:**
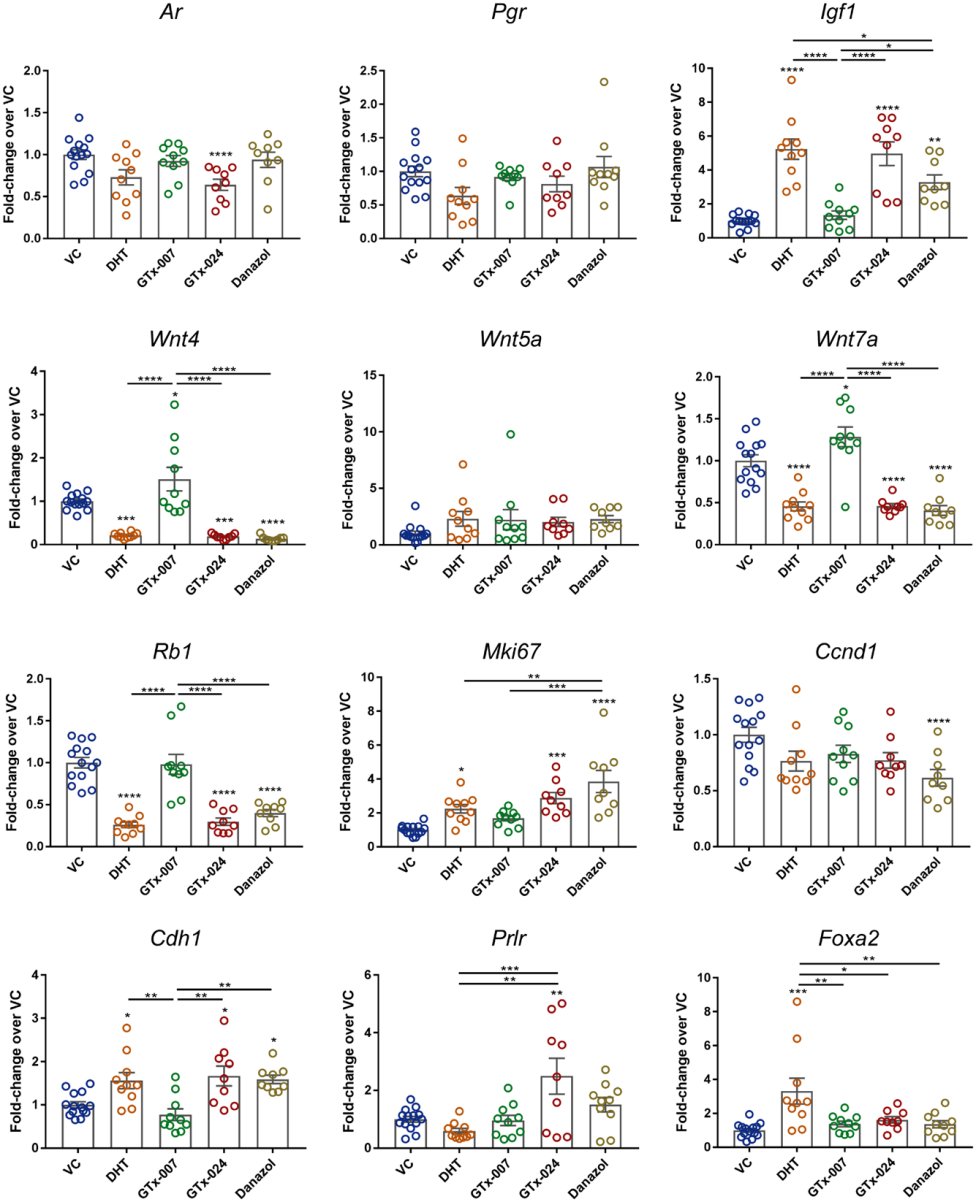
Whole-uterus gene expression changes of proteins involved in proliferation and stromal–epithelial cross-talk. Gene expression analysis by RT-qPCR of mRNAs extracted from whole-uterine homogenates of mice treated with AR ligands; individual genes are identified above the relevant results panels. *n* = 10–14/treatment group. One-way ANOVA with Tukey’s multiple comparisons test was used for comparisons between treatment groups. Plain stars (*) indicate comparisons with VC, while stars above lines demonstrate comparisons between indicated treatment groups (*P < 0.05, **P < 0.01, ***P < 0.001, ****P < 0.0001). DHT, dihydrotestosterone; VC, vehicle control.

mRNAs encoding proteins implicated in epithelial growth and stromal–epithelial cross-talk were measured in uterine tissue homogenates (endometrium plus myometrium) to compare the impact of SARMs and Danazol to that of DHT (Fig. 5). The results demonstrated clearly that at the concentrations administered, the impact of GTx-024 and Danazol on mRNA levels of analysed candidate genes mirrored that of DHT. These included a significant upregulation of the putative androgen-regulated gene *Igf1*, a stromal-derived growth factor that is a key factor in stromal–epithelial cross-talk. Notably, although immuno-expression of AR was increased by GTx-0024 (Fig. 2), mRNA expression was reduced (Fig. 5), highlighting different impacts on protein stability and mRNA turnover. In addition, GTx-024 and Danazol both induced a significant reduction in the uterine expression of *Wnt4* and *Wnt7a*, consistent with previously reported DHT-mediated effects (Simitsidellis *et al.* 2016). The significant increase in the percentage of proliferating cells in the uteri of mice treated with DHT, GTx-024 and Danazol was reflected by a significant upregulation of *Mki67* and downregulation of *Rb1*, together with an increase in *Cdh1* (E-cadherin). Some differences in the effects of GTx-024, Danazol and DHT were also noted, with significant upregulation of *Foxa2* only detected in DHT-treated mice, *Prlr* (prolactin receptor) only increased following treatment with GTx-024 and reduced expression of *Ccnd1* (cyclin D1), that encodes a protein implicated in G1/S phase transition, only detected in the Danazol-treated group.

Whereas other analyses had failed to detect any significant impact of treatment with GTx-007 on tissue morphology or gland number (Figs 1 and 4) analysis of gene expression highlighted increased expression of mRNAs encoded by *Wnt4* and *Wnt7a*, a result that was in marked contrast to the impact of GTx-024, which reduced the expression of the same mRNAs.

## Discussion

The uterus is an androgen-target organ but the effects of SARMs, a new class of nonsteroidal drugs that are being tested as therapies to treat women with breast cancer, urinary incontinence or muscle wasting, on endometrial function have received little attention to date. In this study, we have investigated the uterine-specific effects of two SARMs (GTx-007 and GTx-024), compared their impact to those of the potent endogenous AR agonist DHT and to Danazol, a synthetic drug which is in clinical use but is reported to have androgenic side effects. We conducted the study using a previously established mouse model (Simitsidellis *et al.* 2016), which was designed to mimic the steroid-depleted uterine environment of postmenopausal women, because this is a key target group for therapies related to restoration of muscle function.

The results obtained demonstrate that GTx-024 and Danazol in the mouse uterus largely mirror the impact of DHT, whereas GTx-007, at the supraphysiological dose tested, induced minimal uterine alterations. The significant increase in the uterine weight normalised to body weight of mice treated with GTx-024 is consistent with a rat study by Hoffmann *et al.* (Hoffmann *et al.* 2019). Treatment with DHT and Danazol significantly increased uterine weight compared to VC treatment, as previously reported in studies using either ovariectomised or intact mice (Benghuzzi & England 1995, Zhang *et al.* 2004, Simitsidellis *et al.* 2016) and rats (Lohiya & Arya 1981, Nantermet *et al.* 2005). In the current study, despite the significant increases in the uterine weight of mice induced by DHT (~60 mg), GTx-024 (~47 mg) and Danazol (~46 mg) compared to VC (~22 mg), uterine weight values were still considerably lower than those of intact aged-matched female C57BL/6J mice (~70 mg). This demonstrates these AR ligands can induce restoration of the steroid-depleted uterus, but that this increase in uterine weight does not exceed that of the normal cycling uterus. Notably, two other SARMs, MK-0773 and TFM-4AS-1, both of which bind AR *in vitro*, can induce only modest weight gains in the uterus of ovariectomised rats after 24 days of treatment (Schmidt *et al.* 2010). In addition, the uterus has an outer layer, the myometrium which is made up of smooth muscle cells and in the context of this study provided an additional endpoint for the impact of the ligands on smooth muscle. Our previous studies have documented expression of AR in mouse myometrium (Makieva *et al.* 2016) and in this study, DHT, GTx-024 and Danazol all significantly increased myometrial area.

In the endometrium, GTx-024 exhibited a trophic effect, increasing the area of both the stromal and epithelial compartments, a result consistent with increased percentage of Ki67-positive nuclei in both cell types. While the mechanistic basis for these changes was not studied, it is notable that GTx-024, in common with DHT, significantly increased the expression of *Igf1*. IGF1 acts as an anabolic growth factor in skeletal muscle, stimulating the PI3K/Akt signalling pathway to increase protein synthesis, reduce protein degradation and increase muscle mass (Timmer *et al.* 2018). In the endometrium, IGF1 is a stromal-derived growth factor that binds to the IGF1 receptor on epithelial cells, indirectly mediating the proliferative effects of 17β-oestradiol on epithelial cells (Zhu & Pollard 2007). Consistent with GTx-024 and DHT acting by binding to AR to increase the expression of *Igf1*, androgen response elements (AREs) have been documented on the promoter of the *IGF1* gene in humans (Wu *et al.* 2007). Notably, IGF1 is also upregulated in women with PCOS, a condition often associated with hyperandrogenism (Shafiee *et al.* 2016). A recent publication using the SARM S-42 (Patent No. 5789874, Japan) reported an increase in the weight of the levator ani muscle in rats but failed to detect any change in *Igf1* mRNA when it was used to treat cultured mouse C2C12 myoblasts (Muta *et al.* 2019). Notably, we have previously demonstrated that muscle-resident AR-positive fibroblasts could mediate indirect effects of both GTx-024 and DHT on the levator ani muscle (Dubois *et al.* 2015), highlighting the importance of studying the impact of SARMs on intact tissue as well as isolated cells.

Using the 7-day treatment regime, we noted broadly similar uterine impacts of GTx-024 and Danazol, including significant changes in uterine weight and architecture, although in contrast to GTx-024, Danazol had no significant impact on AR protein levels. With some exceptions, the general consensus is that androgens tend to downregulate the expression of their receptor at the transcriptional level (Quarmby *et al.* 1990, Shan *et al.* 1990, Prins & Woodham 1995, Yeap *et al.* 1999), but stabilise the AR protein (Krongrad *et al.* 1991, Yeap *et al.* 1999) and our results with GTx-024 are consistent with this.

To date, the majority of clinical trials assessing the efficacy of SARMs have used GTx-024, reflecting some of the promising results obtained in preclinical studies, with several focusing on improving muscle function, including that in postmenopausal women (ten trials listed). Some phase II trials have reported promising findings regarding the therapeutic potential of GTx-024, including a statistically significant improvement in total lean body mass and physical function at 3 mg/day for 86 days in postmenopausal women, without reported impacts on sebum production or hair growth (Dalton *et al.* 2011). The phase III clinical trials (POWER1 and POWER2) assessing efficacy of GTx-024 in the prevention and treatment of muscle wasting of non-small-cell lung cancer patients failed to demonstrate significant improvement in physical function, despite significant increases in lean body mass (Crawford *et al.* 2016) (the results of the POWER trials are not yet in published form; clinical trial number NCT01355484). A recent phase II trial which focused on assessing safety and tolerability in postmenopausal women suffering from stress incontinence (*n* = 129) was terminated in October 2018 citing lack of efficacy (NCT03566290), which is of concern for the future of SARMs usage for this common clinical manifestation.

The GTx-007 compound was developed as a treatment for muscle wasting and benign prostatic hyperplasia, but the impacts of the compound on females remain largely unexplored. In one study, 0.1–3 mg/day of GTx-007 were administered to ovariectomised female rats for 120 days, with the authors reporting the doses were based on unpublished pilot data. Results were compared to those obtained using 1 mg/day of DHT (Kearbey *et al.* 2007). The authors reported that GTx-007 had a dose-dependent increase in body weight, lean mass and bone strength. In our study, we did not detect any impact of GTx-007 on body weight or myometrial area, with GTx-007 also displaying minimal uterine effects. In castrated male rats, GTx-007 shows dose-dependent effects in the levator ani muscle; however, GTx-007 is only a partial agonist in the prostate and seminal vesicles, restoring them to 33.8 and 28.2% of intact animals, respectively (Yin *et al.* 2003). Based on our findings, we speculated this might be the case in the uterus, with GTx-007 acting as a partial agonist, but this warrants further investigation.

Gene expression analysis revealed changes in the expression of *Wnt4* and *Wnt7a* in the uteri of treated mice. Wnt proteins play a key role in stromal–epithelial interactions within the uterus, with studies in mice demonstrating expression of *Wnt4* predominantly in the endometrial subluminal stromal compartment and *Wnt7a* in the luminal epithelium (Miller *et al.* 1998). Wnts in the uterus are involved in tissue patterning during postnatal organ development and regulate uterine gland formation, a process termed adenogenesis, with *Wnt4* and *Wnt7a* uterine-knockout mice exhibiting absence of glands due to defective postnatal endometrial epithelial differentiation accompanied by defects in fertility and disrupted expression of genes involved in decidualisation of the endometrium, such as *Hoxa10* and *Lif* (Dunlap *et al.* 2011, Franco *et al.* 2011). In our previous study, we noted a significant decrease in *Wnt4* and *Wnt7a* uterine expression in response to DHT treatment (Simitsidellis *et al.* 2016), a finding that was replicated here and mirrored by treatment with GTx-024 and Danazol. In contrast, treatment with GTx-007 induced a modest but significant upregulation of *Wnt4* and *Wnt7a* in the uteri of treated mice.

Our study was designed to detect impacts on the mouse uterus and had a number of limitations: (1) dosage of treatments was supraphysiological, (2) only one time point was selected for analysis of samples and (3) differences in the impacts of high-dose androgens on uterine architecture between rodents and primates have been reported. (1) For this study, we selected doses of AR ligands previously shown to induce significant uterine changes in mice and rats but that do not reflect their physiological or therapeutic concentrations (Nantermet *et al.* 2005, Ivanga *et al.* 2009, Simitsidellis *et al.* 2016). However, a study by Hoffmann *et al.* demonstrated that even a dose as low as 0.4 mg/kg/day of GTx-024 can elicit a significant increase in uterine wet weight of ovariectomised mice, restoring uterine weight to that of intact mice if treatment continues for 5 weeks (Hoffmann *et al.* 2019), suggesting that the trophic effects of androgens in the rodent uterus are not just a result of high androgen concentrations. (2) We previously reported that treatment of ovariectomised mice with DHT induces an early proliferative response in the uterus at 24 h of treatment, followed by a late uterotrophic phenotype after 7 days of treatment (Simitsidellis *et al.* 2016). In the current study, only the 7-day time point was used for comparative analysis. (3) Importantly, the majority of studies have reported long-term androgen administration at high doses to women and transmen can induce endometrial atrophy (Chadha *et al.* 1994, Perrone *et al.* 2009) without stimulating endometrial proliferation (Zang *et al.* 2007, Wood *et al.* 2009, Simitsidellis *et al.* 2018). However, a recent report on 94 transmasculine persons given testosterone demonstrated that there was persistence of endometrial activity in 67% of the subjects, highlighting the potential for variation in the response of individuals to high doses of androgens (Grimstad *et al.* 2019). In the current study as in others, administering androgens to ovariectomised rodents, treatment with DHT, GTx-024 or Danazol increased the percentage of Ki67-positive cells in the stroma and had a variable impact on epithelial cell proliferation. Our data suggest caution should be exercised when extrapolating rodent uterotrophic effects of androgens to humans and highlights the need for further investigations using different models such as human endometrial tissue explants for the evaluation of SARMs.

We included Danazol, a synthetic derivative of testosterone which is reported to be an effective treatment for endometriosis-associated pain in this study as a comparator to the SARMs and in anticipation that the latter might be used to treat women with endometriosis. Danazol’s mechanism of action is complex, but it is reported to both directly and indirectly reduce ovarian steroidogenesis so that in women with endometriosis both eutopic and ectopic endometrium becomes inactive (Selak *et al.* 2007). While Danazol continues to be prescribed to some women with endometriosis for which other therapies have failed, it is not recommended for women wishing to become pregnant, due to side effects associated with its androgenic activity (https://bnf.nice.org.uk/drug/danazol.html). In our model, Danazol induced an uterotrophic response which was similar to that of DHT a result consistent with reports that it can bind with high affinity to AR.

To date, there have been no reports of adverse androgenic side effects of GTx-024 in women, but these would need to be revisted in future studies should the SARM be evaluated in trials for treatment of women with endometriosis. Notably, our findings and those of others, have demonstrated that in rodents while treatment with DHT or GTx-024 can induce an increase in uterine weight, this only leads to a restoration of the uterine weight of steroid-depleted animals to that of intact animals. New SARMs are still in development, but there is limited information about their use in women. One exception is GSK2881078, which showed promising results in a phase Ib trial, with doses of up to 0.75 mg/day tested for their impact on muscle weakness (Neil *et al.* 2018). This compound was found to have a long half-life in women and lean mass was shown to increase, with women being much more sensitive to lower doses than men highlighting potential sex differences in response.

In summary, administration of the SARM GTx-024 resulted in uterotrophic changes that mimicked those of DHT whereas GTX-007, a SARM with a similar but not identical structure, had little impact. Both SARMs exhibit similar binding to AR in cell-based models highlighting the importance of tissue-based analysis. The development of SARMs with differing impacts on uterine function may offer new therapeutic opportunities for treatment of disorders associated with muscle wasting in women. Based on these results, it is clear that further studies are required to inform choice of therapeutic doses that can be evaluated in clinical trials.

## Supplementary Material

Supplementary Table 1. Details of primers and probes

## Declaration of interest

The authors declare that there is no conflict of interest that could be perceived as prejudicing the impartiality of the research reported.

## Funding

This work was supported by a Medical Research Council Programme grant MR/N024524/1.

## Author contribution statement

P T K S, I S and D A G designed the study; I S, A E-Z, O K and E O’F carried out the experimental work; I S analysed the data; I S, D A G and P T K S wrote the manuscript.
