# Supplementary material for: Selective androgen receptor modulators (SARMs) have specific impacts on the mouse uterus
Source: J Endocrinol. 2019 Jul 18;242(3):227–39. doi: 10.1530/JOE-19-0153 (PMC6690265; doi:10.1530/JOE-19-0153)
Supplement: Supplementary Table 1. Details of primers and probes [file supplementary_table_1.pdf]

Gene Name (*abbreviation*)	Accession Code	Primer Sequences	Primer Position	UPL Probe
Androgen Receptor (*Ar*)	NM_013476	ccagtcccaattgtgtcaaa	1513-1532	58
		tccctggtactgtccaaacg	1584-1603	
Progesterone Receptor (*Pgr*)	NM_008829.2	tgcacctgatctaatcctaaatga	2948 - 2971	17
		ggtaaggcacagcgagtagaa	2994 - 3014	
Insulin-like growth factor 1 (*Igf1*)	ENSMUST00000095360.4	agcagccttccaactcaattat	295 - 316	34
		gaagacgacatgatgtgtatctttatc	345 - 371	
Wingless-related MMTV integration site 4 (*Wnt4*)	ENSMUST00000045747.4	ctggactccctccctgtctt	319 - 338	62
		atgcccttgtcactgcaaa	409 - 427	
Wingless-related MMTV integration site 5A (*Wnt5a*)	ENSMUST00000063465.4	acgcttcgcttgaattcct	565 - 583	55
		cccgggcttaatattccaa	650 - 668	
Wingless-related MMTV integration site 7A (*Wnt7a*)	ENSMUST00000032180.6	cgctcatgaacttacacaataacg	793 - 816	78
		acaggagcctgacacaccat	868 - 887	
Retinoblastoma 1 (*Rb1*)	ENSMUST00000022701.6	gagctcatgagagaccgaca	2237 - 2256	93
		caccttgcagatgccataca	2283 - 2302	
Antigen identified by monoclonal antibody Ki 67 (*MKi67*)	NM_001081117.2	gctgtcctcaagacaatcatca	1753 - 1774	80
		ggcgttatcccaggagact	1805 - 1823	
Cyclin D1 (*Ccnd1*)	ENSMUST00000093962.4	tttctttccagagtcatcaagtgt	870 - 893	72
		tgactccagaagggcttcaa	928 - 947	
Cadherin 1 (*Cdh1*)	ENSMUST00000167688	atcctcgccctgctgatt	2282 - 2299	18
		accaccgttctcctccgta	2325 - 2343	
Prolactin receptor (*Prlr*)	ENSMUST00000124470.1	tggctttgaagggttatagcat	1083 - 1104	31
		cagttcttcagacttgcccttc	1171 - 1192	
Forkhead box A2 (*Foxa2*)	ENSMUST00000047315.3	aagtagccaccacacttcagg	1581 - 1601	32
		tgtggcccatctatttaggg	1632 - 1651	

Supplementary Table 1. Details of primers and probes
